# LncRNA Profile Study Reveals a Three-LncRNA Signature Associated With the Pathological Complete Response Following Neoadjuvant Chemotherapy in Breast Cancer

**DOI:** 10.3389/fphar.2019.00574

**Published:** 2019-05-28

**Authors:** Ying Zeng, Guo Wang, Cheng-Fang Zhou, Hai-Bo Zhang, Hong Sun, Wei Zhang, Hong-Hao Zhou, Rong Liu, Yuan-Shan Zhu

**Affiliations:** ^1^Department of Clinical Pharmacology, Xiangya Hospital, Central South University, Changsha, China; ^2^Hunan Key Laboratory of Pharmacogenetics, Institute of Clinical Pharmacology, Central South University, Changsha, China; ^3^Engineering Research Center of Applied Technology of Pharmacogenomics, Ministry of Education, Changsha, China; ^4^National Clinical Research Center for Geriatric Disorders, Changsha, China; ^5^Department of Pharmacy, Fujian Provincial Hospital, Provincial Clinical College, Fujian Medical University, Fuzhou, China; ^6^Department of Medicine, Weill Cornell Medical College, New York, NY, United States

**Keywords:** biomarker, breast cancer, long non-coding RNA, pathological complete response, PAM50 subtype

## Abstract

**Background:**

The purpose of this study is to develop an effective but concise long non-coding RNA (lncRNA) expression signature that can predict response to neoadjuvant chemotherapy for breast cancer (BC) patients.

**Methods:**

lncRNA expression profiling in 1102 BC patients from Gene Expression Omnibus datasets was analyzed using lncRNA-mining approach. The association between lncRNA signature and pathological complete response (pCR) was analyzed using logistic regression model in the training set (GSE25066, *n* = 488). Validation was performed in independent testing datasets, GSE20194, GSE20271, GSE22093, and GSE23988 (*n* = 614). Bonferroni method was employed for multiple testing corrections. Cell proliferation assay and Western blot assay were performed to evaluate the cell viability and protein expression level, respectively.

**Results:**

Three lncRNAs (AK291479, U79293, and BC032585) have been identified to be significantly associated with pCR in the training dataset (Bonferroni *p*-value < 0.05). Expression signature with these lncRNAs was predictive of pCR in the training (AUC = 0.74) and testing (AUC = 0.72) datasets. Weighted gene co-expression network analysis and gene functional annotation suggest that the three lncRNAs were involved in cell cycle process. To confirm the functional significance of the identified lncRNAs, BC032585 was selectively silenced using RNA interference. Knockdown of BC032585 lncRNA significantly promoted cell resistance to multiple anticancer-drugs through upregulating MDR1 expression in breast cancer cells.

**Conclusion:**

These results suggest that lncRNAs such as BC032585 might be involved in chemotherapeutic response in breast cancer patients, and the three-lncRNA signature identified in the present study may serve as a useful biomarker for the selection of responsive breast cancer patients in neoadjuvant chemotherapy.

## Introduction

Although several targeted agents are implicated in the therapy of breast cancer (BC), traditional cytotoxic chemotherapy remains a mainstay of treatment (von Minckwitz and Martin, 2012; Untch et al., 2014). Neoadjuvant chemotherapy, initially used for the treatment of locally advanced and inoperable tumor, becomes an established therapeutic option for operable BC (Kaufmann et al., 2012). Neoadjuvant chemotherapy improves the outcome of patients treated with surgery (Tabchy et al., 2010; Hatzis et al., 2011). After treatment, response to neoadjuvant chemotherapy is evaluated histologically in the surgical specimen. Pathological complete response (pCR), defined as the complete disappearance of invasive tumor cells in breast and axillary lymph nodes, to neoadjuvant therapy is an effective predictor of tumor progression (Hatzis et al., 2011; von Minckwitz and Martin, 2012), and patients whose tumors show pCR have a better clinical outcome compared to those with residual disease (RD) in the tumor (Bonadonna et al., 1998; Symmans et al., 2007; Rastogi et al., 2008). Unfortunately, there is a considerable proportion of high risk BC patients who do not respond to neoadjuvant chemotherapy (Carey et al., 2007; Untch et al., 2011). Therefore, it is of clinical interest to identify useful biomarker(s) to predict pCR to neoadjuvant chemotherapy, aiming to stratify patients for an optimal therapy. Studies over the years have indicated that clinic-pathological features including histologic grade and estrogen receptor (ER) status (Rouzier et al., 2005b; Huober et al., 2010), and gene expression profiles related to cell proliferation (Silver et al., 2010) and cell cycle (Witkiewicz et al., 2014) are associated with pCR. Tumor prognostic expression values based on the Gene Expression Grade Index (GGI) (Sotiriou et al., 2006), Oncotype Dx (Paik et al., 2004) and Gene70 signature (van ’t Veer et al., 2002) are positively correlated with the pCR probability (Gianni et al., 2005; Liedtke et al., 2009). However, due to the heterogenicity of BC, different BC molecular subtypes classified through PAM50 signatures respond differently to chemotherapy (Rouzier et al., 2005a; Parker et al., 2009).

Accumulating studies have suggested that dysregulated long non-coding RNAs (lncRNAs) were associated with tumorigenesis and progression in a variety of human cancers (Mercer et al., 2009; Gibb et al., 2011). LncRNAs, RNA transcripts range from 200 nucleotides to multiple kilobases in length, lack significant protein-coding capacity (Lipovich et al., 2010). Studies over the years have demonstrated that some dysregulated lncRNAs may contribute as tumor suppressors, while others may serve as proto-oncogenes and/or drivers of metastatic transformation in BC (Mourtada-Maarabouni et al., 2009; Gupta et al., 2010; Silva et al., 2011). It has been shown that lncRNA *HOTAIR* is overexpressed in BC, which might be associated with poor prognosis and high probability of metastasis (Gupta et al., 2010). The overexpression of lncRNA *LSINCT5* is associated with BC cell proliferation and tumor development (Silva et al., 2011). On the other hand, overexpression of lncRNA *GAS5* (growth arrest-specific transcript 5) in MCF-7 BC cells induces apoptosis and growth arrest (Mourtada-Maarabouni et al., 2009). These data collectively suggest that lncRNAs may play a critical role in tumor development and progression and be utilized as biomarker(s) for tumor diagnosis and prognosis.

As the data mining method of repurposing publicly available microarray datasets for lncRNA expression is well-developed (Du et al., 2013; Gellert et al., 2013), new cancer biomarkers for prognosis prediction by making full use of the re-annotated previous microarray datasets have been identified (Hu et al., 2014; Zhou et al., 2016), and lncRNA signatures have been developed to predict overall survival (Meng et al., 2014) and metastasis-free survival (Sun J. et al., 2015) of BC patients. Recently, an lncRNA signature composed of 36 lncRNAs has been reported to serve as a biomarker for predicting pCR to neoadjuvant chemotherapy in breast cancer while the biological functions of these lncRNAs have not been explored (Wang et al., 2017).

To identify an effective but concise prognostic lncRNA biomarker for pCR to neoadjuvant chemotherapy in BC patients, we have mined the available gene expression microarray data from the Gene Expression Omnibus (GEO) and profiled the lncRNA expression data in the present study. By using logistic regression analysis, we identified a three-lncRNA signature that was associated to pCR to neoadjuvant chemotherapy. Moreover, we demonstrated the *BC032585*, one of the three lncRNAs, was directly linked to BC cell sensitivity to chemotherapy. These results suggest that a simple three-lncRNA signature may effectively serve as a predictive biomarker for pCR to neoadjuvant chemotherapy in breast cancer.

## Materials and Methods

### Microarray Data Processing and LncRNA Profile Mining

Five datasets that contain genome-wide gene expression profiling data by using pretreatment biopsies from patients receiving neoadjuvant chemotherapy and corresponding clinical data were download from the GEO databases^1^. After removal of the samples without pathological drug response information, a total of 1102 patients were analyzed, including 488 from GSE25066 (Hatzis et al., 2011), 278 from GSE20194 (Shi et al., 2010), 178 from GSE20271 (Tabchy et al., 2010), 97 from GSE22093 (Iwamoto et al., 2011), and 61 from GSE23988 (Iwamoto et al., 2011). Primary analysis was conducted on dataset GSE25066. The other four datasets were combined and utilized for validation. The demographic characteristics of patients were listed in Table 1.

**Table 1:** Patients’ characteristics of the five datasets.

Characteristics	Training dataset	Validating dataset
**Sample size**	488	614
**Age, years**		
≤50	268	316
>50	220	296
Unknown	0	2
**Histologic grade**		
1	29	32
2	172	213
3	252	306
Unknown	35	63
**Nodal status**		
Positive	334	385
Negative	154	180
Unknown	0	49
**ER**		
Positive	285	338
Negative	197	275
Unknown	6	1
**Response**		
pCR	99	130
RD	389	484
**Neoadjuvant Therapy Regimens**		
TA	488	0
FAC	0	184
TFAC	0	430

*ER, estrogen receptor; TA, taxane and anthracycline; TFAC, taxane, 5-fluorouracil, anthracycline, and cytoxan; FAC, 5-fluorouracil, anthracycline, and cytoxan. pCR, pathological complete response; RD, residual disease.*

The raw CEL files were downloaded from GEO, all gene expression data were generated with Affymetrix U133A gene chips and normalized with RMA algorithm using the ‘affy’ R package. GATExplorer was used to process microarrays for gene expressions of lncRNAs (Risueno et al., 2010). GATExplorer provides a series of R packages called ncRNA Mapper, including the probes that are not assigned to mRNAs but mapped to a database for human non-coding RNA sequences derived from RNAdb (Pang et al., 2007). Each lncRNA should include no less than three probes mapping in the corresponding ncRNA entity. Expressions were calculated according to the re-mapping annotation file (ncrnamapperhgu133acdf) (Risueno et al., 2010).

### Molecular Classification of BC Subtypes

Breast cancer patients were divided into basal-like, luminal A, human epidermal growth factor receptor 2 (HER2) enriched, luminal B, and normal molecular subtypes based on the PAM50 algorithm (Parker et al., 2009) via the “genefu” R package. Samples classified into basal-like, HER2+, luminal A, and luminal B subtypes were included in subtype-based analysis.

### Statistical Analysis of Bioinformatic Data

Statistical computations were performed using the R statistical software version 3.1.0 (R Core Team, 2014) with related packages.

A univariate logistic regression analysis was performed to assess the association between the continuous expression level of each lncRNA and pCR in the training dataset. Expressions of five lncRNAs were identified to be strongly correlated with pCR (Bonferroni *p*-value < 0.01). These five lncRNAs were further analyzed using a multivariable logistic regression model and only three lncRNA expression were found to be significantly associated with pCR (*p*-value < 0.05). For the selection of most predictive model, we evaluated and compared the predictive ability of logistic regression and other four machine learning techniques, namely artificial neural network, regression tree, multivariate adaptive regression splines and Bayesian additive regression trees. The models generated by logistic regression and artificial neural network were outperformed the other approaches in the validating datasets according to the ROC analysis (Supplementary Figure S1). We therefore selected the logistic regression model since it is easier to be understood and implemented in the clinics. A predictive score was then computed according to the formula:

$$predictive\;score\;(PS)=\sum_{i=1}^{n}\left(Exp_{i}^{*}Coe_{i}\right)$$

Where, *n* devotes for the number of prognostic lncRNAs in the model, *Exp*_i_ is the expression level of lncRNA_i_, and *Coe*_i_ is the estimated regression coefficient of lncRNA_i_ in the *i* multivariable logistic regression model. Patients with higher predictive scores are expected to have higher probability of pCR. Furthermore, multivariate logistic analyses were conducted to test whether the predictive score was independent of ER status, pathological grade and PAM50 molecular subtype.

We used receiver operating characteristic (ROC) curves to compare the sensitivity and specificity of molecular signatures used for the prediction of pCR. The area under the receiver operating characteristic curve (AUC) was calculated using the R-package ROCR. The differences between AUCs were tested with *z*-statistical method via the R-package pROC. The performance of the three-lncRNA predictive score developed in this study was compared to other available signatures, including GGI (Sotiriou et al., 2006), Gene70 (Paik et al., 2004) and Oncotype Dx (van ’t Veer et al., 2002). The scores of GGI, Gene70, and Oncotype DX, calculated with “genefu” R package, were utilized as predictors for drawing ROC curves.

### Co-expression Module Detection and Functional Annotation

Co-expression modules were identified with weighted gene co-expression network analysis (WGCNA) (Zhang and Horvath, 2005). The co-expressed relationships between the prognostic lncRNAs and module eigenvalues (MEs) of the modules were computed using Pearson’s correlation coefficients. Gene ontology (GO) biological process enrichment analyses of the modules co-expressed with prognostic lncRNAs were performed to predict the biological function of prognostic lncRNAs via the DAVID annotation tool^2^ with the functional annotation clustering option.

Before WGCNA analysis, all gene expression data were normalized with the RMA algorithm using the ‘affy’ R Bioconductor package. Probe sets without known gene symbols were filtered and probe-level expression profiles were converted into gene-based expressions through probe merging with the collapseRows function (Miller et al., 2011). We adjusted the gene expression levels of entire dataset, which consists of five datasets for potential batch effects, with the ComBat algorithm (Johnson et al., 2007). WGCNA (Zhang and Horvath, 2005) was performed with the “wgcna” R package (Langfelder and Horvath, 2008). A co-expression network was constructed based on 3,600 genes selected based on the following criteria: (i) 5,000 genes with the highest expression variance across the data set; and (ii) the 3,600 genes with the highest degree of co-expression (based on k.total of WGCNA) from the 5,000 genes. To summarize the relationship between all possible pairs of the selected genes, a Pearson’s correlation coefficient was calculated. The correlation matrix was raised to the power 5 and thus producing a weighted network, k.total of a gene was defined as the sum of its adjacency with all other genes for network generation. The topological overlap measure (TOM) was utilized to measure the co-expression similarity for each gene pair from the weighted network. This advanced co-expression measure considers not only the relationship between two genes with each other, but also the extent of their shared connections across the weighted network. We used a hierarchical clustering with dissimilarity based on 1-TOM to produce a hierarchical clustering tree of genes. Modules were identified on the dendrogram using the Dynamic Tree Cut algorithm with a height cutoff of 0.95 and a gene number cutoff of 30 (Liu et al., 2017a). To assess the potential correlation of gene modules with the expression levels of lncRNAs, a summary profile of each module, called the ME that corresponds to the first principal component of the module was calculated. The WGCNA method has been described in detail by Zhang and Horvath (2005).

### Reagents

Doxorubicin (DOX) was purchased from Sigma-Aldrich (St. Louis, MO, United States) and paclitaxel (PTX) from Meilunbio (Dalian, China). Drugs were dissolved in 100% dimethyl sulfoxide (DMSO) as stock solutions. Dilutions for all drug treatments were made extemporaneously in culture medium, so that the final concentration of DMSO never exceeded 0.1% (v/v).

### Cell Culture

Human breast cancer MDA-MB-231 cells and MCF-7 were obtained from the American Type Culture Collection (ATCC, Manassas, VA, United States). Cells were routinely maintained in RPMI-1640 medium (Gibco, Grand Island, NY, United States) containing 10% FBS (BI Technologies, Fullerton, CA, United States), 20 mM L-glutamine (Sigma-Aldrich), 100 U/ml of penicillin (BI Technologies) and 100 μg/ml of streptomycin (BI Technologies) at 37°C in a 5% CO2-95% air humidified atmosphere. The medium was changed every other day.

### Transfection of siRNAs

Three small-interfering RNA (siRNA-1, -2, -3) against *BC032585* gene and a scrambled control small interfering RNA (siRNA) were purchased from RiboBio (Guangzhou, China). The transfection of siRNAs was conducted as previously described (Zhu et al., 2017). Briefly, approximately 3 × 10^5^ cells suspended in 2 ml experimental medium [a mixture of phenol red-free RPMI-1640 medium supplemented with 5% charcoal-dextran-treated FBS (BI Technologies) and 20 mM L-glutamine] were added in each well of 6-well plate. After incubated overnight, 245 μl Opti-MEM medium containing 7.5 μl Lipofectamine RNAiMAX was mixed with a 245 μl Opti-MEM medium containing 10 μl siRNA, and the mixture added to 1.5 ml experimental medium was dripped into each well of 6 well plate. The final concentration of siRNA was 100 nM, a common concentration used in cell culture study (Kothari et al., 2013; Pi et al., 2017). After 24 h incubation at 37°C, the medium was discarded, and the cells were incubated with fresh experimental medium. The sequences of siRNAs were summarized in Supplementary Table S1. The knockdown efficiency was tested by quantitative RT-PCR at 48–72 h after transfection.

### RNA Extraction and Quantitative Real Time (qRT)-PCR

Total RNA extraction and qRT-PCR were performed as previously described (Sun H. et al., 2015). Briefly, total cellular RNA was isolated from harvested cells using the Total RNA Kit II (Omega Bio-Tek, Inc., Norcross, GA, United States) according to the manufacturer’s instruction. First-strand cDNA was generated by reverse-transcription using random primers with the Prime Script RT reagent Kit with gDNA Eraser (TaKaRa BIOTECHNOLOGY, Dalian, China) in a 20 μl reaction containing 0.5–1 μg of total RNAs. The real-time PCR was performed on LightCycler 480 Sequence Detection System using the SYBR Green Realtime PCR Master Mix assay kit (TaKaRa). Reaction parameters were: 95°C for 30 s; then 95°C for 5 s, 55°C for 1 min, and 72°C for 30 s with a cutoff of 45 cycles. Relative gene expression was calculated using the 2^-(ΔΔCT)^ method with β-actin as a reference gene. The primers used were: BC032585, 5′-GCTCTGACAATGTTGTGCTGG-3′ and 5′-GAGTGCTCAAAGTCACACGC-3′; AK291479, 5′-TGACT CTGTGGTTCATTCTGGT-3′ and 5′-CCATCCCCAAGTCAG GAACC-3′; U79293 5′-CTTCTGCTGCTGCTTGGAGT-3′ and 5′-AAGCTCGCCACTCATGACAG-3′; β-actin, 5′-TCAAGATCATTGCTCCTCCTGAG-3′ and 5′-ACATCTGCTGGAAG GTGGACA-3′.

### Cell Proliferation Assay

Cell proliferation assay was performed to detect cell viability using a cell proliferation assay Kit from Promega (Madison, WI, United States) as described previously (Sun H. et al., 2015). Briefly, cells transfected for 24 h with either control or *BC032585* siRNA were passaged to 96-well plates at 5 × 10^3^ cells/well in experimental medium and grown at 37°C for 24 h before drug administration. The cells were then treated with 100 μl of fresh experimental medium containing various concentrations of doxorubicin, paclitaxel or both for 48 h as indicated in each experiment. At the end of experiment, the viable cell number were determined using the Promega assay kit according to the manufacturer’s instruction, and the optical absorbance was determined at 490 nm in a microplate reader. The viability ratio was calculated according to the following formula: the viability ratio = [(the absorbance of experimental group - the absorbance of blank group)/(the absorbance of untreated group - the absorbance of blank group)] × 100%.

### Western Blotting

The level of MDR1 was determined using Western blotting as we previously described (Sun H. et al., 2015). Briefly, total proteins were extracted from cells treated with 100 nM siRNA for 48 and 96 h, and quantified using a BCA Protein Assay Kit following the manufacturer’s instruction (TianGEN, China). A total of 50 μg proteins was denatured, loaded into an 8% SDS-PAGE, electrophoresed and transferred to PVDF membranes. The membrane was blocked in TBST buffer containing 5% non-fat milk for 2 h at room temperature, and then washed with TBST buffer and incubated with a specific primary antibody against MDR1 (1:1000; ab170904, Abcam, United States) or β-actin (1:5000; AC-15, Sigma, United States) at 4°C for 16 h. Following the corresponding secondary antibody incubation for 1 h at room temperature, the signal was visualized using the enhanced chemiluminescence detection system and quantitated under the Bio-Rad ChemiDoc MP system. β-actin was used as a loading control. Each experiment was repeated at least three times.

### Statistical Analysis of Experiment

Statistical analysis was carried out using GraphPad Prism 6 applying Student’s *t*-test for RT-qPCR or western blot comparisons (mRNA or protein expression levels) or 2-way ANOVA test for cell viability comparison. All statistical tests were two-sided and *p* < 0.05 was considered statistically significant.

## Results

### A Three-LncRNA Signature Is Identified to Be Associated With pCR in BC Patients

**FIGURE 1:**
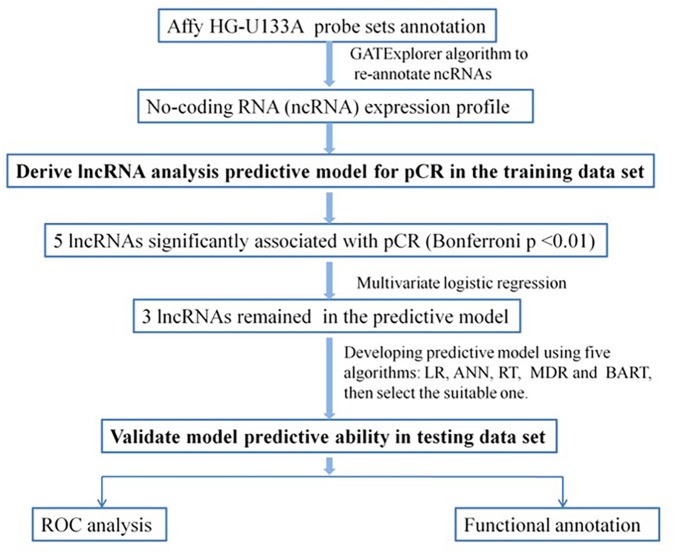
Diagram of the study. Develop predictive model for pCR and validate the efficiency of the lncRNA signature to predict pCR. LR, logistic regression; ANN, artificial neural network; RT, regression tree; MDR, multivariate adaptive regression splines; BART, Bayesian additive regression trees; pCR, pathological complete response; ROC, receiver operating characteristic.

As summarized in the workflow diagram (Figure 1), all initial analyses were performed in the training set and validated in the test set. The training set with 488 BC patients was utilized for the detection of potential lncRNAs that were associated with pCR of chemotherapy. By subjecting the lncRNA expression data of the test series to univariate logistic regression, we identified a set of five lncRNAs that were significantly correlated with patients’ pCR (Bonferroni *P* < 0.05; Supplementary Table S2). Three of the five lncRNAs remained significant correlation with pCR following the analysis by multivariable logistic regression model (Table 2). A positive coefficient in regression analysis for lncRNA *AK291479* and *BC032585* was obtained, indicating that an elevated expression of this lncRNA is associated with a high probability of pCR. In contrast, a negative coefficient for lncRNA *U79293* was observed, indicating that a high expression of *U79293* is associated with a low probability of pCR.

**Table 2:** Association of three lncRNAs with pCR in breast cancer patients treated with neoadjuvant chemotherapy in the training dataset (*n* = 488).

Position	Gene symbol	Univariate analysis			Multivariate Logistic regression		
		OR	95% CI	*P*-value	Bonferroni p value	Coefficient	*P*-value
chrX: 104072568-104076212	*AK291479*	2.27	1.74–2.99	2.87 × 10^-9^	2.00 × 10^-6^	0.5297	4.39 × 10^-4^
chr15: 71293150-71294925	*U79293*	0.19	0.10–0.35	5.13 × 10^-7^	3.58 × 10^-4^	-1.1409	7.83 × 10^-4^
chr9: 37883335-37884282	*BC032585*	4.02	2.14–7.64	1.75 × 10^-5^	1.22 × 10^-2^	0.8039	2.48 × 10^-2^

**FIGURE 2:**
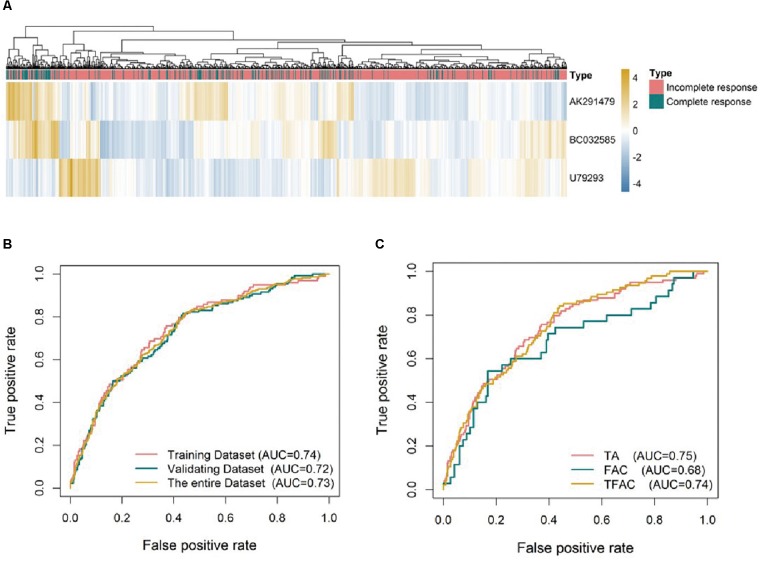
Unsupervised clustering heatmaps and ROC curves for the three lncRNA signature. **(A)** Heatmaps based on 3 lncRNAs (rows) of patients with breast cancer (columns) in the whole datasets (*n* = 1102). Red and blue indicate high and low expression, respectively. ROC curves assess the accuracy of the lncRNA signatures in different datasets **(B)** and for breast cancer patients treated with different chemotherapy regimens: TA, taxane and anthracycline; TFAC, taxane; 5-fluorouracil, anthracycline, and cytoxan; and FAC, 5-fluorouracil, anthracycline, and cytoxan **(C)**. True positive rate represents module sensitivity, whereas false positive rate is one minus the specificity.

In order to choose the most suitable algorithms for predictive model, ROCs for logistic regression, artificial neural network, regression tree, multivariate adaptive regression splines and Bayesian additive regression trees were generated as shown in Supplementary Figure S1. Although the predictive values of regression tree and Bayesian in the training analysis were better than others, the predictive ability using models of logistic regression and artificial neural network (AUC = 0.73) was outperformed the other approaches in the validating dataset (Supplementary Figure S1B). We selected the logistic regression model for subsequent analysis as it is easy to be understood and implicated in clinical practice. Based on the expression of the three identified lncRNAs, a formula was generated for the prediction of pCR: Predictive score = 0.5297^∗^ (expression level of *AK291479*) +0.8039^∗^ (expression level of *BC032585*) -1.1409^∗^ (expression level of *U79293*). This predictive score was able to successfully distinguish between pCR and residual disease (RD) with an AUC value of 0.74, 0.72, and 0.73 in the training, validating and entire dataset, respectively (Figure 2A,B). The predictive value of this lncRNA signature was variant in cohorts treated with different chemotherapeutic regimens. The AUC values of the predictive score were 0.75, 0.74, and 0.68 for patients treated with TA (taxane and anthracycline), TFAC (taxane, 5-fluorouracil, anthracycline, and cytoxan) and FAC (5-fluorouracil, anthracycline, and cytoxan), respectively (Figure 2C), suggesting that this lncRNA signature may be more valuable in predicting anthracycline-based chemotherapeutic response. However, the clinical significance of this predictive score remains to be validated in future clinical trials.

### The Three-LncRNA Signature Is an Independent Predictor of pCR in BC Patients

To ascertain whether this three-lncRNA signature is an independent predictor of pCR in BC patients, we first identified that lncRNA predictive score, histological grade, ER status and PAM50 subtypes were associated with pCR using univariate logistic regression analysis (Table 3). Furthermore, multivariate logistic regression analysis revealed that the lncRNA predictive score was an independent predictor of pCR adjusted by grade, ER status and PAM50 molecular subtype in both the training (OR = 1.60, 95% CI = 1.06–2.44, *p* = 2.65 × 10^-2^) and validating cohort (OR = 1.83, 95% CI = 1.27–2.67, *p* = 1.33 × 10^-3^) as shown in Table 3. In other word, the prognostic ability of the three-lncRNA signature is significant even after accounting for clinicopathologic variables (grade, ER status and PAM50 molecular subtype).

**Table 3:** Logistic regression models for pathological complete response in the training and testing datasets.

	Univariate model			Multivariate model		
	OR	95% CI	*P*-value	OR	95% CI	*P*-value
**Training set**					
lncRNA predictive score	2.72	2.06–3.64	4.49 × 10^-12^	1.60	1.06–2.44	2.65 × 10^-2^
**Grade (ref = 1)**					
2	2.10	0.39–39.00	4.84 × 10^-1^	0.50	0.07–10.30	5.54 × 10^-1^
3	12.32	2.56–221.60	1.44 × 10^-2^	1.51	0.20–31.29	7.25 × 10^-1^
**ER (ref = negative)**					
Positive	0.22	0.14–0.36	8.38 × 10^-10^	0.82	0.35–1.87	6.41 × 10^-1^
**PAM 50 subtypes (ref = Basal-like)**					
Her2+	0.43	0.16–0.98	5.83 × 10^-2^	0.50	0.13–1.53	2.57 × 10^-1^
Luminal A	0.06	0.02–0.14	4.51 × 10^-9^	0.26	0.06–0.95	5.12 × 10^-2^
Luminal B	0.34	0.16–0.65	1.96 × 10^-3^	0.92	0.32–2.69	8.85 × 10^-1^
**Testing set**					
lncRNA predictive score	2.69	2.09–3.51	6.44 × 10^-14^	1.83	1.27–2.67	1.33 × 10^-3^
**Grade (ref = 1)**					
2	3.21	0.63–58.67	2.63 × 10^-1^	1.48	0.27–27.67	7.13 × 10^-1^
3	13.95	2.93–250.25	1.00 × 10^-2^	2.75	0.51–51.32	3.42 × 10^-1^
**ER (ref = negative)**					
positive	0.19	0.12–0.29	1.11 × 10^-13^	0.41	0.20–0.85	1.52 × 10^-2^
**PAM 50 subtypes (ref = Basal-like)**					
Her2+	0.97	0.49–1.87	9.18 × 10^-1^	1.38	0.63–2.99	4.15 × 10^-1^
Luminal A	0.05	0.01–0.13	4.60 × 10^-7^	0.30	0.06–1.05	8.27 × 10^-2^
Luminal B	0.28	0.17–0.44	3.84 × 10^-8^	1.02	0.48–2.16	9.56 × 10^-1^

### The Three-LncRNA Signature Is Equivalent to Other Molecular Signatures in Predicting pCR in BC Patients

**FIGURE 3:**
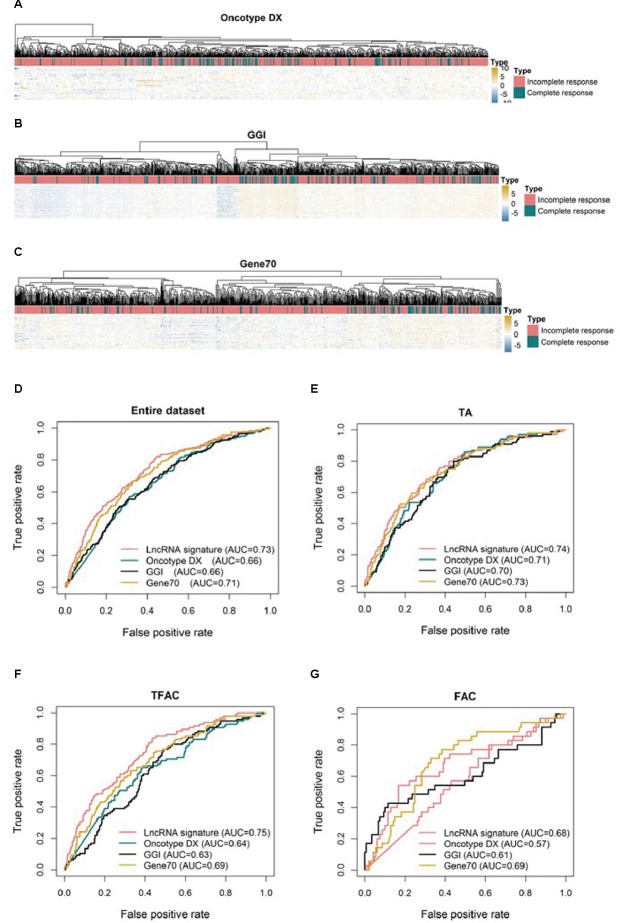
Unsupervised clustering heatmaps and ROC curves for publically available signatures. Heatmaps based on genes (rows) of patients with breast cancer (columns) for the Oncotype DX **(A)**, GGI **(B)**, and Gene70 **(C)** signatures. Red and blue indicate high and low expression, respectively. ROC curves assess the accuracy of the three-lncRNA, Oncotype DX, GGI and Gene70 signatures for all patients **(D)**, patients treated with TA (**E**, taxane and anthracycline), TFAC (**F**, taxane, 5-fluorouracil, anthracycline, and cytoxan) and FAC (**G**, 5-fluorouracil, anthracycline, and cytoxan). True positive rate represents module sensitivity, whereas false positive rate is one minus the specificity.

To compare the three-lncRNA signature to other molecular signatures in predicting pCR, ROC analyses were performed to evaluate the sensitivity and specificity of the lncRNA signature and three published tests, Oncotype DX (Figure 3A), GGI (Figure 3B), and Gene70 (Figure 3C) in predicting pCR. Overall, all four signatures showed predictive power in distinguishing pCR from RD as their AUC values were above 0.5 (Figure 3D). However, the three-lncRNA signature (AUC = 0.73) was significantly superior to GGI and Oncotype DX (AUC = 0.66, *p* < 0.001) and equivalent to Gene70 (AUC = 0.71) as shown in Figure 3D. In the TFAC chemotherapy group (*n* = 430), the performance of the lncRNA signature was outperformed GGI and Oncotype DX (*p* < 0.05) but not Gene70. However, in the cohorts received chemotherapy regimen of TA (*n* = 488) or FAC (184), we found no significant difference (*p* > 0.05) between our defined lncRNA signature and the three published tests (Figure 3E,G), as demonstrated by the AUC values, due to the small sample size (184 in FAC).

### Functional Annotation

**FIGURE 4:**
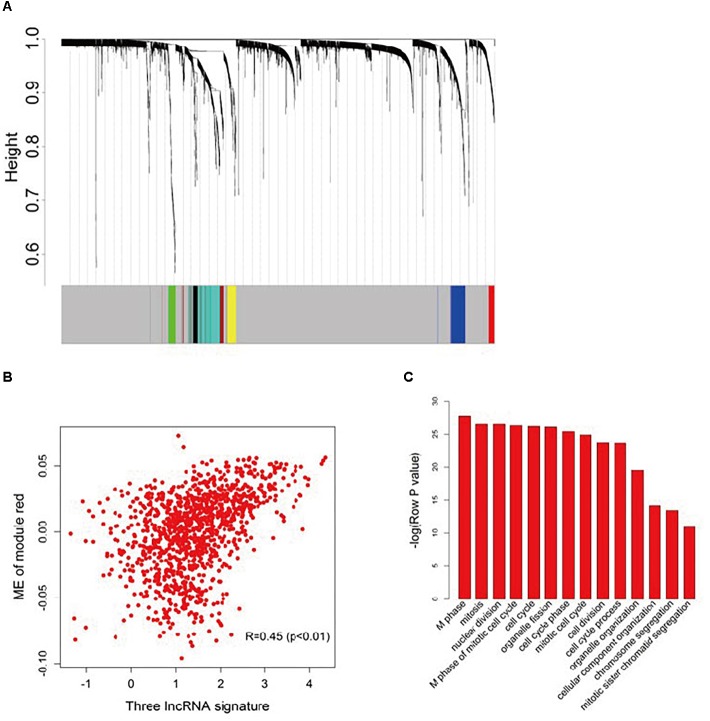
Functional annotation of the three lncRNA signatures. **(A)** Hierarchical cluster analysis dendrogram was utilized to identify co-expression clusters with the gene profiler from the dataset with 1,102 patients with breast cancer treated with chemotherapy. In the picture, branches are presented as the co-expression modules with highly interconnected genes with different colors to indicate module assignment. **(B)** Scatter plot between the three-lncRNA signature and ME of module red. Significant positive correlation relationship between them (*R* = 0.45). **(C)** GO enrichment analysis for the 45 genes comprising the red modules presented multiple processes. The original significance reported by DAVID for GO biological processes were transformed to “–log (*p*-value)” for plotting.

To further investigate the potential biological roles of the three prognostic lncRNAs, the co-expressed relationships between the three-lncRNA signature with the seven co-expression modules (Figure 4A) identified by WGCNA were accessed via Pearson’s correlation coefficients in the entire dataset (Supplementary Table S3). The module-red expression, which composes of 45 genes (Supplementary Table S4), was highly correlated with the predictive score generated from the three-lncRNA signature (*R* = 0.45, *p* < 0.001, Figure 4B). Moreover, Pearson correlation analysis of each dataset revealed a significant negative correlation between BC032585 and ABCB1 gene expression (Supplementary Figure S2). GO function enrichment analysis of the module red by using the whole human genome as the background suggested that module red was significantly enriched in 34 GO terms (Bonferroni *p* < 0.05, Supplementary Table S5). Among them, 14 terms were related to cell cycle/proliferation as shown in Figure 4C, suggesting that the three-lncRNA signature might be involved in the regulation of cell proliferation through modulating protein-coding gene expression.

### Effect of BC032585 LncRNA on Drug Sensitivity

**FIGURE 5:**
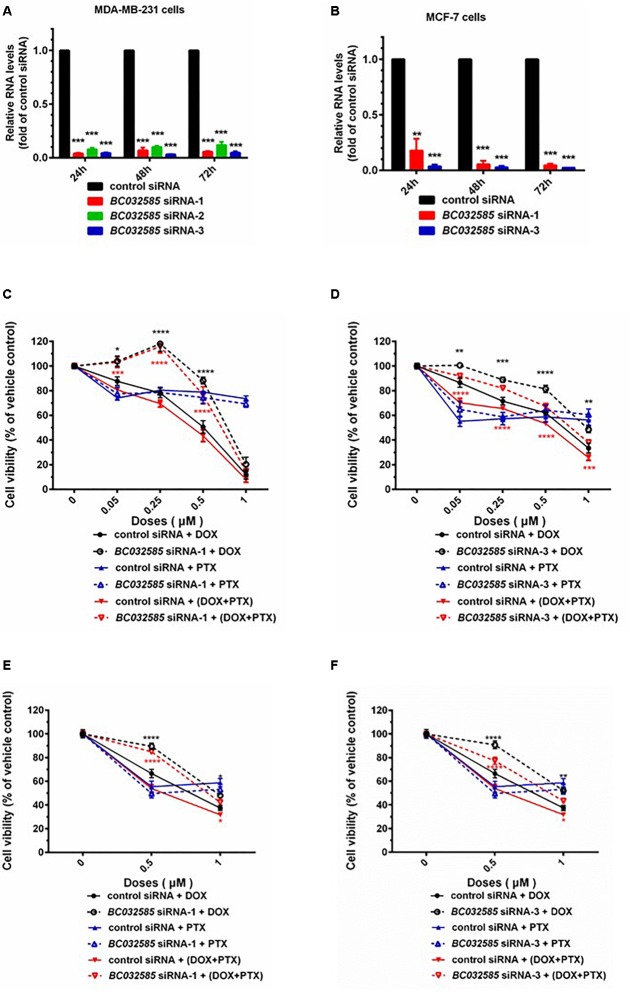
Knockdown of *BC032585* lncRNA increases cell resistance to chemotherapeutic agents in MDA-MB-231 and MCF-7 breast cancer cells. **(A,B)** MDA-MB-231 and MCF-7 cells were transfected with a specific *BC032585* siRNA (*BC032585* siRNA –1, 2, 3, and 100 nM), or a negative control siRNA (control siRNA) for 24, 48, and 72 h. The transfected cells were then harvested for quantification of *BC032585* lncRNA by real-time PCR. The RNA levels were expressed as fold of corresponding controls, and the data are mean ± SD of three independent experiment. ^∗∗∗^*p* < 0.001 compared to the corresponding controls. **(C,D)** MDA-MB-231 cells were transfected with either *BC032585* siRNAs or control siRNA for 24 h, and then plated in 96-well plates and treated with various doses of DOX alone or in combination with 0.05 μM PTX for 48 h. The number of viable cells (cell viability) was determined at the end of treatment and expressed as a percentage of corresponding vehicle control. The data are the mean ± SEM of two or three independent triplicate experiments. ^∗^*p* < 0.05, ^∗∗^*p* < 0.01, ^∗∗∗^*p* < 0.001 compared to corresponding controls. **(E,F)** MCF-7 cells in 96-well plate were transfected with either *BC032585* siRNAs or control siRNA for 48 h and then treated with various doses of DOX alone or in combination with 0.05 μM PTX for another 48 h. The number of viable cells (cell viability) was determined at the end of treatment and expressed as a percentage of corresponding vehicle control. The data are the mean ± SEM of two or three independent triplicate experiments. ^∗^*p* < 0.05, ^∗∗^*p* < 0.01, ^∗∗∗^*p* < 0.001, ^∗∗∗∗^*p* < 0.0001 compared to corresponding controls.

Based on the bioinformatics analysis, we observed that an increased expression of *AK291479* and *BC032585* lncRNA and/or a decreased expression of *U79293* lncRNA was associated with a high probability of pCR following neoadjuvant chemotherapy, i.e., a high sensitivity to chemotherapeutic agent(s). To verify this observation experimentally, we performed *in vitro* study to determine the cell sensitivity to chemotherapeutic agents, taxane and anthracyclines, in MDA-MB-231 (a triple negative BC cell line) and MCF-7 (an ER-positive) BC cells with or without an alteration in *BC032585* lncRNA expression. The *BC032585* lncRNA was selected since it has a relatively high expression in MDA-MB-231 cells compared to the other two lncRNAs (Supplementary Figure S3). As shown in Figure 5, the expression of *BC032585* lncRNA in MDA-MB-231 (Figure 5A) and MCF-7 cells (Figure 5B) was dramatically decreased more than 90% using RNA interference with 3 specific siRNAs. Two of the three siRNAs were selected to knockdown *BC032585* lncRNA level and the transfected cells were exposed to TA agents. After treatment for 48 h with 0.05, 0.1, 0.25, 0.5, and 1 μM DOX alone or in combination with 0.05 μM PTX, MDA-MB-231 cells transfected with *BC032585* siRNA-1 (Figure 5C) or siRNA-3 (Figure 5D) showed a significant resistance to chemotherapy, either DOX alone or in combination with PTX, compared to parallel control siRNA-treated cells. On the other hand, knockdown of *BC032585* had no significant effect on MDA-MB-231 cell sensitivity to PTX treatment (Figure 5C,D). Furthermore, similar alternations in chemo-sensitivity to DOX and PTX treatment were observed in MCF-7 cells when *BC032585* was knocked down (Figure 5E,F). These results collectively suggest that *BC032585* primarily regulates cell sensitivity to DOX but not to PTX.

**FIGURE 6:**
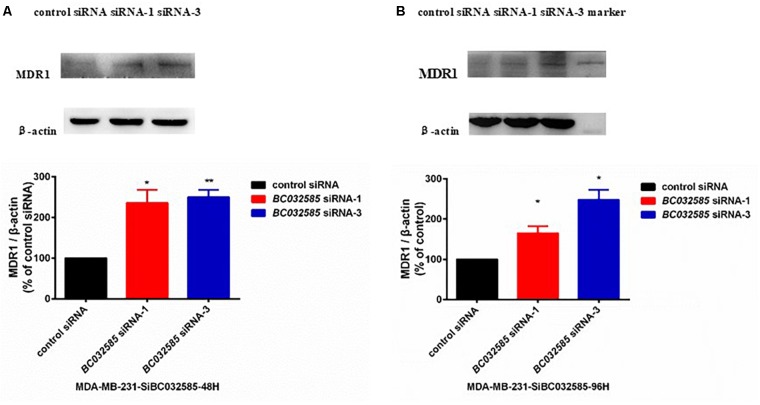
Knockdown of *BC032585* lncRNA increases MDR1 expression in MDA-MB-231 breast cancer cells. **(A,B)** MDA-MB-231 cells were transiently transfected with a specific *BC032585* siRNA (100 nM) or a control siRNA for 48 h **(A)** and 96 h **(B)**. The transfected cells were then harvested for quantification of MDR1 protein levels by Western blot analysis. β-actin was used as an internal loading control. The protein levels were expressed as fold of corresponding controls, and the data are mean ± SD of two or three independent experiments. ^∗^*p* < 0.05, ^∗∗^*p* < 0.01 compared to the corresponding control.

To further investigate the mechanism of how *BC032585* lncRNA modulates the sensitivity of tumor cells to TA treatment, we examined MDR1 protein expression in MDA-MB-231 cells following *BC032585* knockdown as both DOX an PTX are substrates of MDR1, and MDR1 is known to confer resistance to a variety of anticancer agents including doxorubicin and paclitaxel (Abraham et al., 2010; Chen et al., 2016). Furthermore, there was a significant negative correlation between *BC032585*and ABCB1 expression (Supplementary Figure S2). Western blot analysis revealed that knockdown of *BC032585* by two specific siRNAs resulted in a significant elevation of the MDR1 protein level in MDA-MB-231 cells (Figure 6). These results suggest that the effect of *BC032585* lncRNA on chemotherapeutic drug sensitivity is mediated, at least in part, through the upregulation of MDR1 expression in MDA-MB-231 cells.

## Discussion

Accumulating evidence in the study of dysregulated lncRNA expression across various cancer types indicate that lncRNAs play critical roles in tumorigenesis (Zhang et al., 2013) through modifications of multiple cancer related biological processes such as apoptosis, cell cycle regulation and metastasis (Gupta et al., 2010; Cui et al., 2013). Furthermore, these dysregulated lncRNAs could mark the spectrum of tumor progression with a great potential as novel independent molecular biomarkers for the diagnosis and prognosis of cancer (Hu et al., 2014; Meng et al., 2014, Liu et al., 2017b, 2018). Recently, several studies have functionally characterized multiple pathogenesis-related lncRNAs in BC (Mourtada-Maarabouni et al., 2009; Gupta et al., 2010; Silva et al., 2011), but the prognostic value of these lncRNAs has been merely investigated (Meng et al., 2014; Sun J. et al., 2015). Recently, a 36-lncRNA signature has been reported to predict pCR to neoadjuvant chemotherapy in breast cancer patients (Wang et al., 2017), however, it did not perform experimental analysis of the functional significance of any identified lncRNA. To explore the prognostic value of lncRNAs in neoadjuvant chemotherapy, we have utilized microarray probe mining to repurpose the existing human Affymetrix microarray data (platform HG-U133A) and subsequently obtained lncRNA expression profiles of 1102 BC patients from GEO database. Using association analysis between lncRNA expression and pCR in breast cancer patients following neoadjuvant chemotherapy in the training dataset, a three-lncRNA signature has been identified to be independently associated with pCR, which is further confirmed in the validation dataset.

Following multivariable logistic regression analysis, we have revealed that the prognostic value of this three-lncRNA signature was independent of other main prognostic factors including estrogen receptor (ER) status, tumor grade and molecular subtypes. It has been reported that ER-negative and high-grade breast cancers were consistently associated with a better response to chemotherapy (Rouzier et al., 2005b; Kurozumi et al., 2015); and different molecular subtypes of breast cancers based on PAM50 classifier responded differently to neoadjuvant chemotherapy (Parker et al., 2009). Specifically, HER2 positive and basal-like subgroups are associated with the highest rates of pCR (about 45%), whereas the luminal tumors had a pCR rate of only 6% (Rouzier et al., 2005a). In agreement with previous studies, we have observed that ER status, tumor grade and PAM50 molecular subtypes were significantly associated with pCR when evaluated by the univariate logistic regression analysis in both the training and validating datasets (Table 3). Moreover, we have demonstrated through multivariable logistic regression analysis that the three-lncRNA signature is an independent factor in the prediction of pCR in breast cancer patients treated with neoadjuvant chemotherapy (Table 3). Furthermore, we have found that the predictive power of this three-lncRNA signature is equivalent to that of PAM50 (AUC = 0.73), and higher than those of ER status (AUC = 0.69) and tumor grade (AUC = 0.68) as analyzed by ROC (Supplementary Figure S4, *p* < 0.05). In addition, the predictive power, based on the AUC of ROC analysis (Figure 3), of the current three-lncRNA signature is comparable or superior to three previously published molecular signatures: GGI, Gene 70, and Oncotype DX (Figure 3). For patients treated with TFAC (Figure 3F), the AUC of the three-lncRNA signature is higher than those of GGI, Gene70, and Oncotype DX (*p* < 0.05). Meanwhile, compared to the three mRNA-profile based molecular signatures, which require to analyze hundreds of mRNAs, only three lncRNAs need to be quantitated in the current three-lncRNA signature to generate an equivalent or superior efficiency in predicting pCR in breast cancer patients treated with neoadjuvant chemotherapy. Taken together, the three-lncRNA signature identified in our current study may be a simple, efficient and economical biomarker for stratifying breast cancer patients in neoadjuvant chemotherapy.

It should be noted that the biological functions of these three lncRNAs have not been investigated. Interestingly, *AK291479* overlaps with the transcript of thymosin beta-15B (TMSB15B), one of the two isoforms of human thymosin beta 15, TMSB15A and TMSB15B, which show approximately equivalent levels of expression in MCF-7 breast cancer cells (Banyard et al., 2009). Moreover, it has been reported that thymosin beta 15A (TMSB15A) is a predictor of pCR of chemotherapy in triple-negative breast cancer (Darb-Esfahani et al., 2012). WGCNA and gene functional annotation analysis suggest that these three lncRNAs are likely to involve in cell cycle and cell proliferation, cell features related to pCR (Sotiriou et al., 2006).

To experimentally confirm the functional significance of the identified lncRNAs in drug sensitivity, we have studied the effect of *BC032585*, one of the three lncRNAs, on sensitivity to taxane and anthracyclines treatment in MDA-MB-231 and MCF-7 breast cancer cells. As expected, knockdown of *BC032585* lncRNA using specific siRNAs in the cells resulted in a cell resistance to the treatment of doxorubicin and doxorubicin plus paclitaxel, but not paclitaxel alone as accessed by cell viability (Figure 5). These experimental results support our bioinformatic analyses that an increased expression of *BC032585* lncRNA is associated with a high probability of pCR following neoadjuvant chemotherapy (i.e., a high sensitivity to chemotherapy), and the three-lncRNA signature has a better predictive power in anthracycline-based chemotherapeutic response. Since anthracycline is a first-line chemotherapeutic agent for metastatic breast cancer (Gustafson et al., 2005), this three-lncRNA signature may serve as a potential biomarker for personalized chemotherapy in BC patients.

To explore the molecular mechanism of the identified lncRNAs on drug sensitivity, we determined MDR1 expression in MDA-MB-231 breast cancer cells transfected with specific siRNAs against *BC032585*, one of the three lncRNAs. Knockdown of *BC032585* significantly increased the MDR1 expression in MDA-MB-231 cells (Figure 6). As MDR1 is a well-documented membrane drug efflux and responsible for multiple drug resistance including doxorubicin (Abraham et al., 2010; Chen et al., 2016), the current data suggest that the *BC032585*-related drug sensitivity may be mediated, at least in part, through the regulation of MDR1 expression although the molecular mechanism of *BC032585* regulation of MDR1 expression remains to be elucidated. This observation also supports our previous demonstration that lncRNA-regulated MDR1 alteration is a critical pathway mediating chemoresistance in breast cancer cells (Zhu et al., 2017).

How lncRNA *BC032585* regulates MDR1 expression is currently unclear. Previous studies have suggested that lncRNAs may regulate target gene expression through either *cis*- or *trans*-regulation. Xiao et al. (2017) have reported that lncRNA *UCA1* functioned as a competitive endogenous RNA (ceRNA) of *ABCB1* through completive binding of microRNA16 in chronic myeloid leukemia cells. Zhang et al. (2015) have shown that lncRNA *PVT1* could promote MDR1 expression through *mTOR/HIF-1a/P-gp* signaling pathway in gastric cancer cells, and our previous study have demonstrated that lncRNA H19 could regulate MDR1 expression through a *H19-CUL4A-MDR1* pathway in breast cancer cells (Zhu et al., 2017). This data collectively suggests that lncRNA *BC032585* may regulate MDR1 expression through either a *cis*- or *trans*-regulatory mechanism, which is under investigation in our laboratory.

There are multiple limitations in the present study. First, despite a fraction of human lncRNAs (698 out of 20000+) were included in our analysis, GATExplorer was not available for use any more, which was the first system that integrates mapping of probes with genomic contextual views, as well as expression signals at probe level. By far, the most common lncRNA annotation platform is GENCODE, and the version of which is constantly updated. Second, although WGCNA and gene functional annotation analysis suggest that the three lncRNAs may involve in cell cycle and cell proliferation, functional analysis only performed for *BC032585* and the other two lncRNAs, *AK291479* and *U79293*, remain to be investigated. Moreover, the molecular mechanisms of these lncRNAs in modulation of drug sensitivity warrant further investigation, especially those factors associated with this lncRNA signature (Supplementary Table S4). Finally, next-generation sequencing technology has advantages over microarray and has been employed worldwide for molecular diagnostics and identifying predictors of chemosensitivity. Although we have identified and validated this three-lncRNA signature in four independent datasets, its clinical significance and application need further investigation including next-generation sequencing data analysis in prospective clinical trials.

## Conclusion

In summary, via probing and integrating publicly available microarray datasets, we have identified a three-lncRNA signature that is independently associated with pCR in breast cancer patients treated with neoadjuvant chemotherapy. Based on ROC analysis, this signature is comparable or superior to previously published molecular signatures and clinicopathological features in predicting pCR in breast cancer patients treated with neoadjuvant chemotherapy. Moreover, we have demonstrated that one of the three lncRNAs, *BC032585*, plays a significant role in cell sensitivity to chemotherapeutic agents presumably mediated through regulation of MDR1 expression. Taken together, these data indicate that this three-lncRNA signature may serve as a useful biomarker in stratifying advanced breast cancer patients in neoadjuvant chemotherapy, and as potential molecular targets for optimization of breast cancer chemotherapy.

## Author Contributions

YZ performed the experiments, analyzed the data, and wrote the manuscript. RL and Y-SZ designed the study and analyzed the data. All authors critically revised the whole manuscript.

## Conflict of Interest Statement

The authors declare that the research was conducted in the absence of any commercial or financial relationships that could be construed as a potential conflict of interest.

## Footnotes

1 http://www.ncbi.nlm.nih.gov/geo/

2 http://david.abcc.ncifcrf.gov/

## References

[B1] Abraham I. Jain S. Wu C. P. Khanfar M. A. Kuang Y. Dai C. L. (2010). Marine sponge-derived sipholane triterpenoids reverse P-glycoprotein (ABCB1)-mediated multidrug resistance in cancer cells. *Biochem. Pharmacol.* 80 1497–1506. 10.1016/j.bcp.2010.08.001 20696137 PMC2948058

[B2] Banyard J. Barrows C. Zetter B. R. (2009). Differential regulation of human thymosin beta 15 isoforms by transforming growth factor beta 1. *Genes Chromosomes Cancer* 48 502–509. 10.1002/gcc.20659 19296525 PMC2756613

[B3] Bonadonna G. Valagussa P. Brambilla C. Ferrari L. Moliterni A. Terenziani M. (1998). Primary chemotherapy in operable breast cancer: eight-year experience at the milan cancer institute. *J. Clin. Oncol.* 16 93–100. 10.1200/jco.1998.16.1.93 9440728

[B4] Carey L. A. Dees E. C. Sawyer L. Gatti L. Moore D. T. Collichio F. (2007). The triple negative paradox: primary tumor chemosensitivity of breast cancer subtypes. *Clin. Cancer Res.* 13 2329–2334. 10.1158/1078-0432.ccr-06-1109 17438091

[B5] Chen Z. Shi T. Zhang L. Zhu P. Deng M. Huang C. (2016). Mammalian drug efflux transporters of the ATP binding cassette (ABC) family in multidrug resistance: a review of the past decade. *Cancer Lett.* 370 153–164. 10.1016/j.canlet.2015.10.010 26499806

[B6] Core Team R. (2014). *R: A Language and Environment for Statistical Computing*. Vienna: R Foundation for Statistical Computing.

[B7] Cui Z. Ren S. Lu J. Wang F. Xu W. Sun Y. (2013). The prostate cancer-up-regulated long noncoding RNA PlncRNA-1 modulates apoptosis and proliferation through reciprocal regulation of androgen receptor. *Urol. Oncol.* 31 1117–1123. 10.1016/j.urolonc.2011.11.030 22264502

[B8] Darb-Esfahani S. Kronenwett R. von Minckwitz G. Denkert C. Gehrmann M. Rody A. (2012). Thymosin beta 15A (TMSB15A) is a predictor of chemotherapy response in triple-negative breast cancer. *Br. J. Cancer* 107 1892–1900. 10.1038/bjc.2012.475 23079573 PMC3504944

[B9] Du Z. Fei T. Verhaak R. G. Su Z. Zhang Y. Brown M. (2013). Integrative genomic analyses reveal clinically relevant long noncoding RNAs in human cancer. *Nat. Struct. Mol. Biol.* 20 908–913. 10.1038/nsmb.2591 23728290 PMC3702647

[B10] Gellert P. Ponomareva Y. Braun T. Uchida S. (2013). Noncoder: a web interface for exon array-based detection of long non-coding RNAs. *Nucleic Acids Res.* 41:e20. 10.1093/nar/gks877 23012263 PMC3592461

[B11] Gianni L. Zambetti M. Clark K. Baker J. Cronin M. Wu J. (2005). Gene expression profiles in paraffin-embedded core biopsy tissue predict response to chemotherapy in women with locally advanced breast cancer. *J. Clin. Oncol.* 23 7265–7277. 10.1200/jco.2005.02.0818 16145055

[B12] Gibb E. A. Brown C. J. Lam W. L. (2011). The functional role of long non-coding RNA in human carcinomas. *Mol. Cancer* 10:38. 10.1186/1476-4598-10-38 21489289 PMC3098824

[B13] Gupta R. A. Shah N. Wang K. C. Kim J. Horlings H. M. Wong D. J. (2010). Long non-coding RNA HOTAIR reprograms chromatin state to promote cancer metastasis. *Nature* 464 1071–1076. 10.1038/nature08975 20393566 PMC3049919

[B14] Gustafson D. L. Merz A. L. Long M. E. (2005). Pharmacokinetics of combined doxorubicin and paclitaxel in mice. *Cancer Lett.* 220 161–169. 10.1016/j.canlet.2004.09.007 15766591

[B15] Hatzis C. Pusztai L. Valero V. Booser D. J. Esserman L. Lluch A. (2011). A genomic predictor of response and survival following taxane-anthracycline chemotherapy for invasive breast cancer. *JAMA* 305 1873–1881. 10.1001/jama.2011.593 21558518 PMC5638042

[B16] Hu Y. Chen H. Y. Yu C. Y. Xu J. Wang J. L. Qian J. (2014). A long non-coding RNA signature to improve prognosis prediction of colorectal cancer. *Oncotarget* 5 2230–2242. 24809982 10.18632/oncotarget.1895 PMC4039159

[B17] Huober J. von Minckwitz G. Denkert C. Tesch H. Weiss E. Zahm D. M. (2010). Effect of neoadjuvant anthracycline-taxane-based chemotherapy in different biological breast cancer phenotypes: overall results from the gepartrio study. *Breast Cancer Res. Treat.* 124 133–140. 10.1007/s10549-010-1103-9 20697801

[B18] Iwamoto T. Bianchini G. Booser D. Qi Y. Coutant C. Shiang C. Y. (2011). Gene pathways associated with prognosis and chemotherapy sensitivity in molecular subtypes of breast cancer. *J. Natl. Cancer Inst.* 103 264–272. 10.1093/jnci/djq524 21191116

[B19] Johnson W. E. Li C. Rabinovic A. (2007). Adjusting batch effects in microarray expression data using empirical bayes methods. *Biostatistics* 8 118–127. 10.1093/biostatistics/kxj037 16632515

[B20] Kaufmann M. von Minckwitz G. Mamounas E. P. Cameron D. Carey L. A. Cristofanilli M. (2012). Recommendations from an international consensus conference on the current status and future of neoadjuvant systemic therapy in primary breast cancer. *Ann. Surg. Oncol.* 19 1508–1516. 10.1245/s10434-011-2108-2 22193884

[B21] Kothari V. Wei I. Shankar S. Kalyana-Sundaram S. Wang L. Ma L. W. (2013). Outlier kinase expression by RNA sequencing as targets for precision therapy. *Cancer Discov.* 3 280–293. 10.1158/2159-8290.cd-12-0336 23384775 PMC3597439

[B22] Kurozumi S. Inoue K. Takei H. Matsumoto H. Kurosumi M. Horiguchi J. (2015). ER, PgR, Ki67, p27(Kip1), and histological grade as predictors of pathological complete response in patients with HER2-positive breast cancer receiving neoadjuvant chemotherapy using taxanes followed by fluorouracil, epirubicin, and cyclophosphamide concomitant with trastuzumab. *BMC Cancer* 15:622. 10.1186/s12885-015-1641-y 26345461 PMC4562359

[B23] Langfelder P. Horvath S. (2008). WGCNA: an R package for weighted correlation network analysis. *BMC Bioinformatics* 9:599. 10.1186/1471-2105-9-559 19114008 PMC2631488

[B24] Liedtke C. Hatzis C. Symmans W. F. Desmedt C. Haibe-Kains B. Valero V. (2009). Genomic grade index is associated with response to chemotherapy in patients with breast cancer. *J. Clin. Oncol.* 27 3185–3191. 10.1200/JCO.2008.18.5934 19364972 PMC2716940

[B25] Lipovich L. Johnson R. Lin C. Y. (2010). MacroRNA underdogs in a microRNA world: evolutionary, regulatory, and biomedical significance of mammalian long non-protein-coding RNA. *Biochim. Biophys. Acta* 1799 597–615. 10.1016/j.bbagrm.2010.10.001 20951849

[B26] Liu R. Hu R. Zhang W. Zhou H. H. (2018). Long noncoding RNA signature in predicting metastasis following tamoxifen treatment for ER-positive breast cancer. *Pharmacogenomics* 10.2217/pgs-2018-0032 [Epub ahead of print]. 29983093

[B27] Liu R. Zeng Y. Zhou C. F. Wang Y. Li X. Liu Z. Q. (2017a). Long noncoding RNA expression signature to predict platinum-based chemotherapeutic sensitivity of ovarian cancer patients. *Sci. Rep.* 7:18. 10.1038/s41598-017-00050-w 28154416 PMC5428368

[B28] Liu R. Zhang W. Liu Z. Q. Zhou H. H. (2017b). Associating transcriptional modules with colon cancer survival through weighted gene co-expression network analysis. *BMC Genomics* 18:361. 10.1186/s12864-017-3761-z 28486948 PMC5424422

[B29] Meng J. Li P. Zhang Q. Yang Z. Fu S. (2014). A four-long non-coding RNA signature in predicting breast cancer survival. *J. Exp. Clin. Cancer Res.* 33:84. 10.1186/s13046-014-0084-7 25288503 PMC4198622

[B30] Mercer T. R. Dinger M. E. Mattick J. S. (2009). Long non-coding RNAs: insights into functions. *Nat. Rev. Genet.* 10 155–159. 10.1038/nrg2521 19188922

[B31] Miller J. Cai C. Langfelder P. Geschwind D. Kurian S. Salomon D. (2011). Strategies for aggregating gene expression data: the collapserows R function. *BMC Bioinformatics* 12:322. 10.1186/1471-2105-12-322 21816037 PMC3166942

[B32] Mourtada-Maarabouni M. Pickard M. R. Hedge V. L. Farzaneh F. Williams G. T. (2009). GAS5, a non-protein-coding RNA, controls apoptosis and is downregulated in breast cancer. *Oncogene* 28 195–208. 10.1038/onc.2008.373 18836484

[B33] Paik S. Shak S. Tang G. Kim C. Baker J. Cronin M. (2004). A multigene assay to predict recurrence of tamoxifen-treated, node-negative breast cancer. *N. Engl. J. Med.* 351 2817–2826. 15591335 10.1056/NEJMoa041588

[B34] Pang K. C. Stephen S. Dinger M. E. Engstrom P. G. Lenhard B. Mattick J. S. (2007). RNAdb 2.0–an expanded database of mammalian non-coding RNAs. *Nucleic Acids Res.* 35 D178–D182. 10.1093/nar/gkl926 17145715 PMC1751534

[B35] Parker J. S. Mullins M. Cheang M. C. Leung S. Voduc D. Vickery T. (2009). Supervised risk predictor of breast cancer based on intrinsic subtypes. *J. Clin. Oncol.* 27 1160–1167. 10.1200/jco.2008.18.1370 19204204 PMC2667820

[B36] Pi F. Binzel D. W. Lee T. J. Li Z. Sun M. Rychahou P. (2017). Nanoparticle orientation to control RNA loading and ligand display on extracellular vesicles for cancer regression. *Nat. Nanotechnol.* 13 82–89. 10.1038/s41565-017-0012-z 29230043 PMC5762263

[B37] Rastogi P. Anderson S. J. Bear H. D. Geyer C. E. Kahlenberg M. S. Robidoux A. (2008). Preoperative chemotherapy: updates of national surgical adjuvant breast and bowel project protocols B-18 and B-27. *J. Clin. Oncol.* 26 778–785. 10.1200/JCO.2007.15.0235 18258986

[B38] Risueno A. Fontanillo C. Dinger M. E. De Las Rivas J. (2010). GATExplorer: genomic and transcriptomic explorer; mapping expression probes to gene loci, transcripts, exons and ncRNAs. *BMC Bioinformatics* 11:221. 10.1186/1471-2105-11-221 20429936 PMC2875241

[B39] Rouzier R. Perou C. M. Symmans W. F. Ibrahim N. Cristofanilli M. Anderson K. (2005a). Breast cancer molecular subtypes respond differently to preoperative chemotherapy. *Clin. Cancer Res.* 11 5678–5685. 10.1158/1078-0432.ccr-04-2421 16115903

[B40] Rouzier R. Pusztai L. Delaloge S. Gonzalez-Angulo A. M. Andre F. Hess K. R. (2005b). Nomograms to predict pathologic complete response and metastasis-free survival after preoperative chemotherapy for breast cancer. *J. Clin. Oncol.* 23 8331–8339. 10.1200/jco.2005.01.2898 16293864

[B41] Shi L. Campbell G. Jones W. D. Campagne F. Wen Z. Walker S. J. (2010). The microarray quality control (MAQC)-II study of common practices for the development and validation of microarray-based predictive models. *Nat. Biotechnol.* 28 827–838. 10.1038/nbt.1665 20676074 PMC3315840

[B42] Silva J. M. Boczek N. J. Berres M. W. Ma X. Smith D. I. (2011). LSINCT5 is over expressed in breast and ovarian cancer and affects cellular proliferation. *RNA Biol.* 8 496–505. 10.4161/rna.8.3.14800 21532345

[B43] Silver D. P. Richardson A. L. Eklund A. C. Wang Z. C. Szallasi Z. Li Q. (2010). Efficacy of neoadjuvant cisplatin in triple-negative breast cancer. *J. Clin. Oncol.* 28 1145–1153. 10.1200/jco.2009.22.4725 20100965 PMC2834466

[B44] Sotiriou C. Wirapati P. Loi S. Harris A. Fox S. Smeds J. (2006). Gene expression profiling in breast cancer: understanding the molecular basis of histologic grade to improve prognosis. *J. Natl. Cancer Inst.* 98 262–272. 10.1093/jnci/djj052 16478745

[B45] Sun H. Wang G. Peng Y. Zeng Y. Zhu Q. N. Li T. L. (2015). H19 lncRNA mediates 17beta-estradiol-induced cell proliferation in MCF-7 breast cancer cells. *Oncol. Rep.* 33 3045–3052. 10.3892/or.2015.3899 25846769

[B46] Sun J. Chen X. Wang Z. Guo M. Shi H. Wang X. (2015). A potential prognostic long non-coding RNA signature to predict metastasis-free survival of breast cancer patients. *Sci. Rep.* 5:16553. 10.1038/srep16553 26549855 PMC4637883

[B47] Symmans W. F. Peintinger F. Hatzis C. Rajan R. Kuerer H. Valero V. (2007). Measurement of residual breast cancer burden to predict survival after neoadjuvant chemotherapy. *J. Clin. Oncol.* 25 4414–4422. 10.1200/jco.2007.10.6823 17785706

[B48] Tabchy A. Valero V. Vidaurre T. Lluch A. Gomez H. Martin M. (2010). Evaluation of a 30-gene paclitaxel, fluorouracil, doxorubicin, and cyclophosphamide chemotherapy response predictor in a multicenter randomized trial in breast cancer. *Clin. Cancer Res.* 16 5351–5361. 10.1158/1078-0432.ccr-10-1265 20829329 PMC4181852

[B49] Untch M. Fasching P. A. Konecny G. E. Hasmuller S. Lebeau A. Kreienberg R. (2011). Pathologic complete response after neoadjuvant chemotherapy plus trastuzumab predicts favorable survival in human epidermal growth factor receptor 2-overexpressing breast cancer: results from the TECHNO trial of the AGO and GBG study groups. *J. Clin. Oncol.* 29 3351–3357. 10.1200/jco.2010.31.4930 21788566

[B50] Untch M. Konecny G. E. Paepke S. von Minckwitz G. (2014). Current and future role of neoadjuvant therapy for breast cancer. *Breast* 23 526–537. 10.1016/j.breast.2014.06.004 25034931

[B51] van ’t Veer L. J. Dai H. van de Vijver M. J. He Y. D. Hart A. A. Mao M. (2002). Gene expression profiling predicts clinical outcome of breast cancer. *Nature* 415 530–536. 11823860 10.1038/415530a

[B52] von Minckwitz G. Martin M. (2012). Neoadjuvant treatments for triple-negative breast cancer (TNBC). *Ann. Oncol.* 23 (Suppl. 6), vi35–vi39. 10.1093/annonc/mds193 23012300

[B53] Wang G. Chen X. Liang Y. Wang W. Shen K. (2017). A long noncoding RNA signature that predicts pathological complete remission rate sensitively in neoadjuvant treatment of breast cancer. *Transl. Oncol.* 10 988–997. 10.1016/j.tranon.2017.09.005 29096247 PMC5671410

[B54] Witkiewicz A. K. Balaji U. Knudsen E. S. (2014). Systematically defining single-gene determinants of response to neoadjuvant chemotherapy reveals specific biomarkers. *Clin. Cancer Res.* 20 4837–4848. 10.1158/1078-0432.CCR-14-0885 25047707 PMC5286972

[B55] Xiao Y. Jiao C. Lin Y. Chen M. Zhang J. Wang J. (2017). lncRNA UCA1 contributes to imatinib resistance by acting as a ceRNA against mir-16 in chronic myeloid leukemia cells. *DNA Cell Biol.* 36 18–25. 10.1089/dna.2016.3533 27854515

[B56] Zhang B. Horvath S. (2005). A general framework for weighted gene co-expression network Analysis. *Stat. Appl. Genet. Mol. Biol.* 4:17. 10.2202/1544-6115.1128 16646834

[B57] Zhang H. Chen Z. Wang X. Huang Z. He Z. Chen Y. (2013). Long non-coding RNA: a new player in cancer. *J. Hematol. Oncol.* 6:37. 10.1186/1756-8722-6-37 23725405 PMC3693878

[B58] Zhang X. W. Bu P. Liu L. Zhang X. Z. Li J. (2015). Overexpression of long non-coding RNA PVT1 in gastric cancer cells promotes the development of multidrug resistance. *Biochem. Biophys. Res. Commun.* 462 227–232. 10.1016/j.bbrc.2015.04.121 25956062

[B59] Zhou M. Sun Y. Sun Y. Xu W. Zhang Z. Zhao H. (2016). Comprehensive analysis of lncRNA expression profiles reveals a novel lncRNA signature to discriminate nonequivalent outcomes in patients with ovarian cancer. *Oncotarget* 7 32433–32448. 10.18632/oncotarget.8653 27074572 PMC5078024

[B60] Zhu Q. N. Wang G. Guo Y. Peng Y. Zhang R. Deng J. L. (2017). LncRNA H19 is a major mediator of doxorubicin chemoresistance in breast cancer cells through a cullin4A-MDR1 pathway. *Oncotarget* 8 91990–92003. 10.18632/oncotarget.21121 29190892 PMC5696158

