# Supplementary material for: LncRNA Profile Study Reveals a Three-LncRNA Signature Associated With the Pathological Complete Response Following Neoadjuvant Chemotherapy in Breast Cancer
Source: Front Pharmacol. 2019 May 28;10:574. doi: 10.3389/fphar.2019.00574 (PMC6546925; doi:10.3389/fphar.2019.00574)
Supplement: Supplementary file 1 [file Data_Sheet_1.zip › Supplementary materials/Suppl. Tables 1, 3 and Suppl. Figures.docx]

**Supplementary**

**TABLE S1** The sequences of siRNAs used in the study

| **Gene name** | **Product ID** | **Sequences** |
| --- | --- | --- |
| BC032585 | siG160829011611 | GCCACTTCCAATTGACTAA |
| BC032585 | siG160829011623 | GCACTATGGCTGGTTAAAT |
| BC032585 | siG160829011637 | CCACACAGGTGGTAGAAAT |
| Negative control | SiN05815122147 |  |

**TABLE S3** Correlation coefficient between predictive score and MEs of the modules

|  | red | blue | green | yellow | black | brown | turquoise |
| --- | --- | --- | --- | --- | --- | --- | --- |
| Predictive score | 0.45 | -0.02 | 0.16 | -0.18 | 0.23 | 0.08 | 0.07 |

| **A 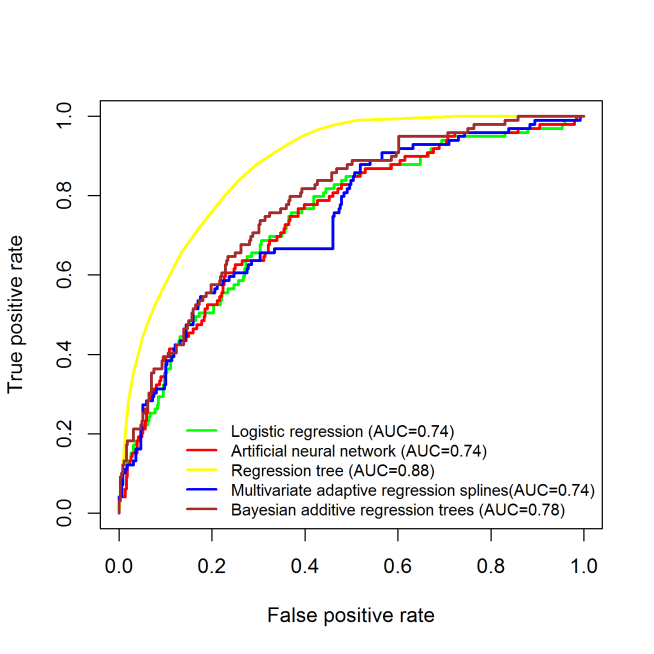** | **B**  **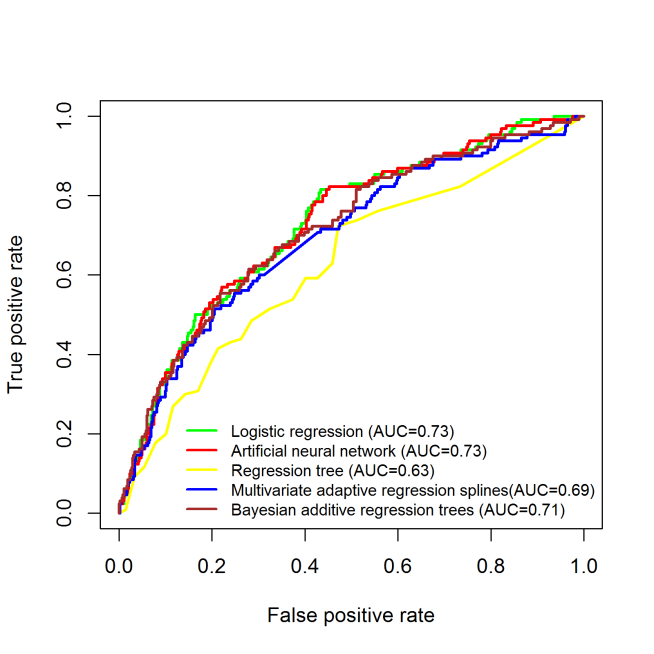** |
| --- | --- |

**FIGURE S1** ROC curves for different predictive algorithms in the training (**A**) and validating (**B**) datasets. Abbreviations: LR: logistic regression; ANN: artificial neural network, RT: regression tree; MDR: multivariate adaptive regression splines; BART: Bayesian additive regression trees.

A. B. C.


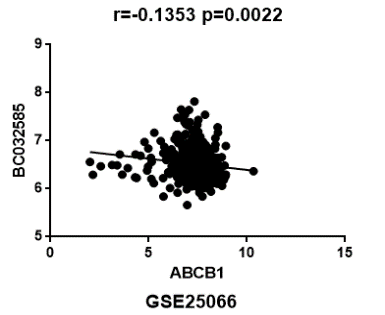

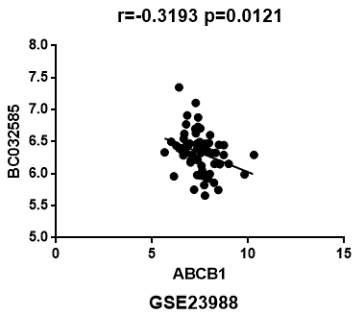

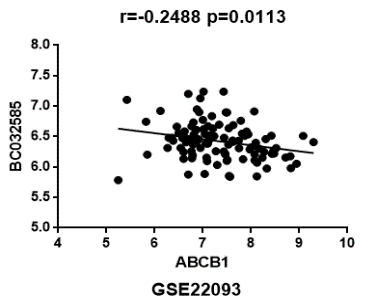


E. F.


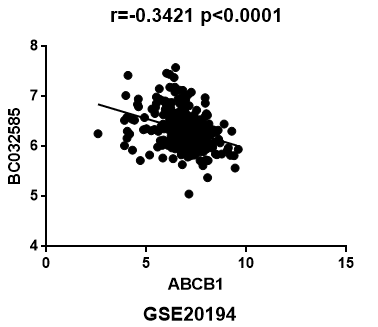

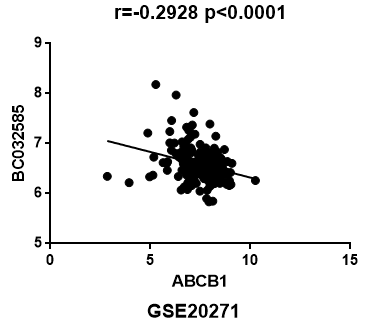


**FIGURE S2** Pearson’s correlation coefficient between BC032585 and ABCB1 in each dataset.





**FIGURE S3** Relative RNA Expression level of *AK291479*, *U79293* and *BC032585* in MDA-MB-231 cells. The data are the means ± SD of three independent experiments. *p<0.05 for *AK291479* vs *U79293*, ***p<0.001 for *BC032585* vs *AK291479* and *U79293*.


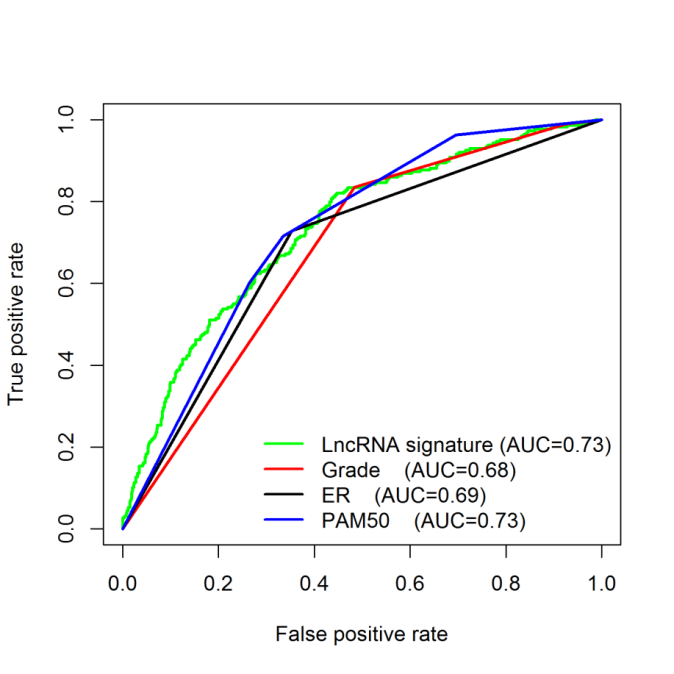


**FIGURE S4** ROC curves assess the accuracy of the three-lncRNA, Grade, ER and PAM50. True positive rate represents module sensitivity, whereas false positive rate is one minus the specificity.
